# Congenital absence of the right carotid artery with a left carotid artery pseudoaneurysm: a case report

**DOI:** 10.3389/fsurg.2025.1702772

**Published:** 2025-11-27

**Authors:** Miaomiao Chen, Junxiang Tang, Muhammad Asad Iqbal

**Affiliations:** 1 Department of Critical Care Medicine, the Fourth Affiliated Hospital of Jiangsu University, Zhenjiang, China; 2 Department of Otorhinolaryngology, First People's Hospital of Zhenjiang, Zhenjiang, China; 3 Department of Ultrasound, Women’s Hospital, Zhejiang University School of Medicine, Hangzhou, China

**Keywords:** neck mass, carotid artery absence, carotid artery pseudoaneurysm, congenital vascular anomaly, carotid revascularization

## Abstract

A unilateral carotid artery pseudoaneurysm combined with a congenital absence of the contralateral internal carotid artery is clinically rare, and the currently available literature is limited. This article reports a case of a 37-year-old male patient with a left carotid artery pseudoaneurysm and congenital absence of the right common and internal carotid arteries, initially presenting with a neck mass. The patient recovered well 2 years after surgery.

## Introduction

The congenital absence of the common or internal carotid artery is extremely rare, with an incidence below 0.01% (1). A carotid artery pseudoaneurysm, often associated with trauma or an infection, poses a life-threatening risk if untreated (2). The coexistence of carotid artery agenesis and a pseudoaneurysm is exceptionally rare, with few cases reported.

## Case report

**Figure 1:**
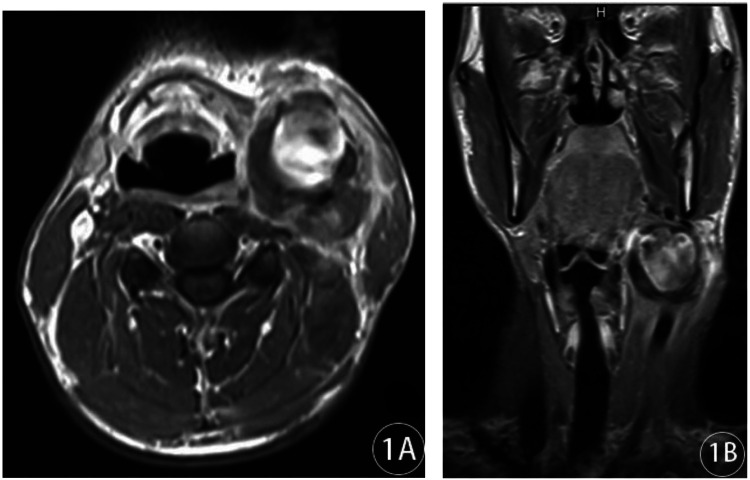
Plain MRI + enhanced MRI of the neck: **(A)** axial and **(B)** coronal.

**Figure 2:**
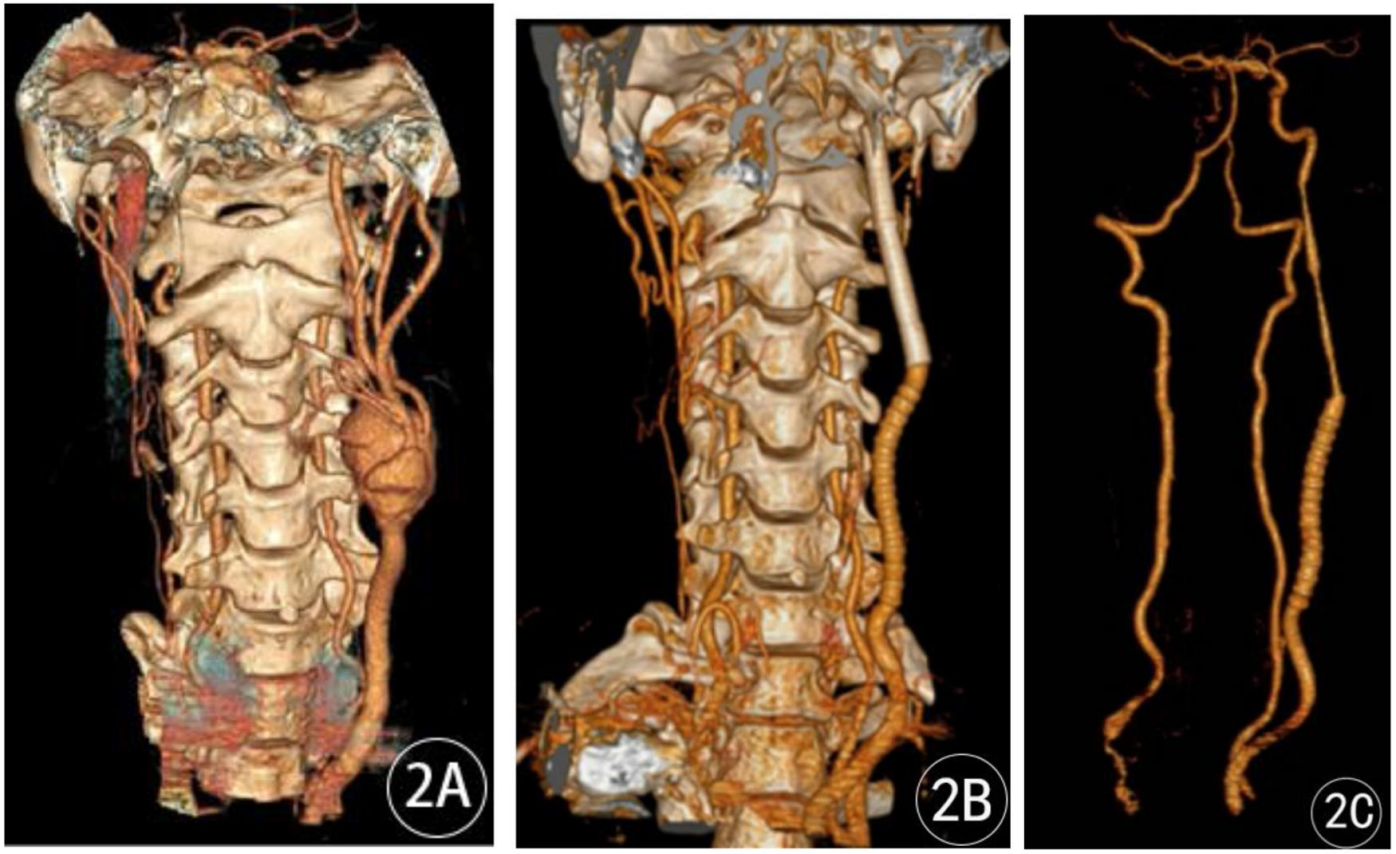
Three-dimensional reconstructions of CTA of the neck: **(A)** before the operation and **(B,C)** 10 days after the operation.

A 37-year-old male patient presented to our hospital on 21 October 2019, complaining of a left-sided neck mass that had been present for a year, had grown significantly larger over a month, and had been accompanied by pain for 2 days. The mass had intermittently increased or decreased in size over the course of the illness, but the patient did not seek medical attention or treatment. Over the previous month, the mass had shown signs of significant enlargement, and over the previous 2 days, he had experienced left-sided neck pain, with a visual analog scale score of 4/10, accompanied by hoarseness and odynophagia, but no dyspnea. The patient had no history of recurrent headaches or dizziness, nor of cerebral ischemia or subarachnoid hemorrhage. The physical examination revealed a soft neck, a neutral trachea, and normally sized thyroid glands. A 4.0 cm × 5.0 cm mass was present at the lateral left mandibular angle, without local redness or swelling. It was pulsating and tender to palpation, and a vascular bruit was audible on auscultation. He had no previous surgical history, did not routinely monitor his blood pressure, and denied any history of hypertension. His highest blood pressure on admission was 180/100 mmHg. Plain and contrast-enhanced MRI of the neck showed a tumor around the left common carotid artery, measuring approximately 43 mm × 47 mm, with unclear boundaries, flow void signals inside, and exudation surrounding it. The common carotid artery and the internal and external carotid arteries were displaced anteriorly, and the adjacent tissues were compressed, with flocculent and uneven enhancement (Figures 1A,B). A three-dimensional reconstruction of computed tomography angiography (CTA) of the neck showed that the left common carotid artery was thickened near the bifurcation and bulged posteriorly, measuring approximately 27.1 mm × 24.8 mm, with obvious enhancement. The left internal carotid artery originated from the top of the raised tumor cavity, with low-density shadows surrounding it. The right internal and common carotid arteries were not visualized, but the external carotid artery was visible and connected to the tortuous vessels on the right side of the neck. Multiple tortuous blood vessels were found in the posterior cervical muscle tissue near the right vertebral artery, which was considered to be collateral circulation (Figure 2A).

The patient underwent routine outpatient examinations and was diagnosed with a left carotid artery pseudoaneurysm preoperatively. Cerebral perfusion was initially assessed through cranial and cervical imaging. A joint consultation between neurosurgeons and vascular surgeons considered the lack of visualization of the right internal and common carotid arteries, which made conventional carotid artery occlusion testing difficult. Furthermore, the left carotid pseudoaneurysm had recently grown in size and could rupture at any time, posing a life-threatening risk. After assessing for any surgical contraindications, a left carotid pseudoaneurysm resection and revascularization were immediately performed. During the operation, the left carotid pseudoaneurysm was resected and replaced with a graft. During the operation, the aneurysm was opened and the cavity revealed a large amount of fresh thrombus and a plasma-like, pale yellow fluid, confirming the pseudoaneurysm diagnosis. Postoperatively, routine symptomatic treatment was administered, including dehydration to reduce intracranial pressure and anticoagulation, antiplatelet, and neurotrophic therapies. On the first postoperative day, the patient developed sudden motor aphasia, accompanied by coarse and fine motor impairment in the right hand and tongue extension with left deviation. He remained alert and had normal muscle tone. An emergency cranial CT scan revealed no significant abnormalities, and a cranial MRI was not performed. A multidisciplinary consultation suspected acute cerebral infarction, and emergency carotid artery graft revascularization was performed. Postoperatively, symptomatic treatment with antiplatelet and anticoagulation therapies, dehydration, intracranial pressure reduction, and expectoration was continued. Follow-up CTA of the carotid and vertebral arteries 10 days after surgery revealed that after resection of the left common carotid artery aneurysm, the right internal and common carotid arteries were not visualized, while the external carotid artery was visible (Figures 2B,C). After discharge, the patient received rehabilitation therapy at a rehabilitation hospital. A follow-up 2 years after surgery showed that the patient had recovered well, with gradual restoration of normal speech and limb motor function.

## Discussion

The congenital absence of the common and internal carotid arteries is rare in clinical practice, and its cause and compensatory mechanism are not yet fully understood (3). Tode et al. found an absence of the internal carotid artery during a physical examination in 1787. As of 2020, there have been fewer than 100 reports of common carotid artery agenesis, and the incidence of internal carotid artery agenesis is reported to be less than 0.01% (4). Cases of a combined absence of the common and internal carotid arteries are even rarer.

The common carotid artery develops embryonically from the third aortic arch and the abdominal aorta. It is the result of the regression of the dorsal aorta between the third and fourth aortic arches (carotid ductus). Before occlusion of the carotid ductus, abnormal regression of the third aortic arch can lead to the absence of the common carotid artery (5). In this case, CTA showed that the right common carotid artery had only a small stump at the origin of the brachiocephalic trunk, the bilateral vertebral arteries were thick, and the external carotid artery originated directly from the brachiocephalic trunk (6). The internal carotid artery develops from the first and third aortic arches and the paired dorsal aorta (7). The absence of the internal carotid artery occurs due to the abnormal regression of the first and third aortic arches (8). The cause of the disease is still unclear.

Clinically, patients without a carotid artery may experience headaches, paroxysmal epilepsy, transient ischemic attacks, and subarachnoid hemorrhage. However, these patients may not have obvious clinical symptoms due to a well-developed collateral circulation. In this case, the patient had no obvious headaches, epilepsy, cerebral ischemia, or subarachnoid hemorrhage, which was considered to be related to compensation by the vertebrobasilar artery system and the posterior communicating artery. Zink et al. (9) reported that carotid artery hypoplasia has been shown to increase the risk of cerebral aneurysms from 2%–4% in the general population to 27.8%. This is likely due to changes in blood flow and velocity due to the internal carotid artery being absent. The local vascular pressure increases and compensatory tortuosity and thickening of the vessels ensure normal cerebral blood supply, which in turn damages the vascular endothelium and induces cerebral aneurysms. This case presented with a left common carotid artery aneurysm in addition to the absence of the right common and internal carotid arteries. The aneurysm showed intermittent changes in size during the course of the disease. Combined with the imaging studies, the possibility of a pseudoaneurysm was considered, suggesting that this aneurysm may have been the result of local vascular reactivity compensation.

This patient presented to our department with a pulsating mass in his neck. Further examination revealed the absence of the common and internal carotid arteries, which is rare in clinical practice. Color Doppler ultrasound can be used as a preliminary screening tool to diagnose the absence of the common carotid artery. When the doctor cannot detect a normal carotid bifurcation, it may indicate the possibility of the absence of the common carotid artery (10). At this point, further digital subtraction angiography (DSA), CTA, or magnetic resonance angiography (MRA) is necessary. Previously, DSA was the gold standard for diagnosing carotid artery agenesis, but due to its high radiation exposure and invasiveness, it is now being replaced by CTA or MRA.

In summary, combined common and internal carotid artery agenesis is a rare clinical condition. Because patients often have increased collateral circulation, most cases may not present with specific clinical manifestations, and in principle, no treatment is required. However, because the incidence of cerebral aneurysms among patients with carotid artery agenesis is much higher than in the general population, those with this condition combined with an aneurysm should be given special attention, and early surgical treatment of the aneurysm is recommended.

After reviewing this case, we recommend that attending physicians should be aware of the following: 1) While a unilateral carotid artery aneurysm combined with congenital absence of the other carotid artery is rare, for patients with a unilateral carotid artery aneurysm, attention should be paid not only to the aneurysm but also to the compensatory status of the contralateral carotid artery. This condition should be considered in the absence of other related medical conditions or when imaging studies reveal no visualization of the vessels; and 2) For patients with a confirmed absence of the carotid artery, perioperative attention must be paid to cerebral perfusion, with timely implementation of appropriate measures and active postoperative anticoagulation and antithrombotic therapies to avoid or minimize complications.

## Data Availability

The original contributions presented in the study are included in the article/Supplementary Material; further inquiries can be directed to the corresponding author.
